# Supplementary material for: FERN – a Java framework for stochastic simulation and evaluation of reaction networks
Source: BMC Bioinformatics. 2008 Aug 29;9:356. doi: 10.1186/1471-2105-9-356 (PMC2553347; doi:10.1186/1471-2105-9-356)
Supplement: Additional file 1 — FERN distribution, Version 1.3. This archive contains the FERN source code and binaries as well as documentation and example models in FernML and SBML. [file 1471-2105-9-356-S1.zip › fern/doc/javadoc/fern/network/sbml/SBMLPropensityCalculator.html]

SBMLPropensityCalculator


---


|  |  |  |
| --- | --- | --- |
| **Overview** \| **Package** \| **Class** \| **Use** \| **Tree** \| **Deprecated** \| **Index** \| **Help** | |  |
| **PREV CLASS**   NEXT CLASS | **FRAMES**    **NO FRAMES**     **All Classes** |  |
| SUMMARY: NESTED \| FIELD \| CONSTR \| METHOD | DETAIL: FIELD \| CONSTR \| METHOD |  |


---


## fern.network.sbml Class SBMLPropensityCalculator

```
java.lang.Object
  fern.network.sbml.SBMLPropensityCalculator
```

**All Implemented Interfaces:**: ComplexDependenciesPropensityCalculator, PropensityCalculator

**Direct Known Subclasses:**: CellDesignerPropensityCalculator

---

``` public class SBMLPropensityCalculator extends Object implements ComplexDependenciesPropensityCalculator ```

Propensity calculator which is used for `SBMLNetwork`s. The propensities are
calculated by using a `MathTree` derived by the MathML representation of the
kinetic law for each reaction.

**Author:**
:   Florian Erhard

---

**Constructor Summary**

`SBMLPropensityCalculator(SBMLNetwork net)`
  
          Creates the `MathTree`s and parses the parameters.


| **Method Summary** | |
| --- | --- |
| `double` | `calculatePropensity(int reaction, AmountManager amount, Simulator sim)`             Calculates the propensity for a reaction given the amounts of the `AmountManager`. |
| `Map<String,Double>` | `getGlobalParameters()`             Gets the global parameters. |
| `List<Integer>` | `getKineticLawSpecies(int reaction)`             Gets the indices of the species that are included in the calculation of the given reaction. |
| `MathTree` | `getMathTree(int reaction)`             Gets the internal representation of the sbml kinetic law. |

**Methods inherited from class java.lang.Object**

`clone, equals, finalize, getClass, hashCode, notify, notifyAll, toString, wait, wait, wait`

**Constructor Detail**

### SBMLPropensityCalculator

```
public SBMLPropensityCalculator(SBMLNetwork net)
```

:   Creates the `MathTree`s and parses the parameters.

    **Parameters:**: `net` - sbml netowrk


**Method Detail**

### getGlobalParameters

```
public Map<String,Double> getGlobalParameters()
```

:   Gets the global parameters.

    :   **Returns:**: global parameters

---


### calculatePropensity

```
public double calculatePropensity(int reaction,
                                  AmountManager amount,
                                  Simulator sim)
```

:   **Description copied from interface: `PropensityCalculator`**
:   Calculates the propensity for a reaction given the amounts of the `AmountManager`.
    If a positive
    value for volume is given, it is assumed that the constants are deterministic rate
    constants and are hence to be transformed to specific reaction rate constants.

    :   **Specified by:**: `calculatePropensity` in interface `PropensityCalculator`
    :   **Parameters:**: `reaction` - index of the reaction: `amount` - AmountManager: `sim` - Simulator **Returns:**: actual propensity of the reaction

---


### getKineticLawSpecies

```
public List<Integer> getKineticLawSpecies(int reaction)
```

:   **Description copied from interface: `ComplexDependenciesPropensityCalculator`**
:   Gets the indices of the species that are included in the calculation of
    the given reaction.

    :   **Specified by:**: `getKineticLawSpecies` in interface `ComplexDependenciesPropensityCalculator`
    :   **Parameters:**: `reaction` - index of the reaction **Returns:**: indices of the species included in the reaction's kinetic law

---


### getMathTree

```
public MathTree getMathTree(int reaction)
```

:   Gets the internal representation of the sbml kinetic law.

    :   **Parameters:**: `reaction` - index of the reaction **Returns:**: a MathTree representation of the kinetic law


---


|  |  |  |
| --- | --- | --- |
| **Overview** \| **Package** \| **Class** \| **Use** \| **Tree** \| **Deprecated** \| **Index** \| **Help** | |  |
| **PREV CLASS**   NEXT CLASS | **FRAMES**    **NO FRAMES**     **All Classes** |  |
| SUMMARY: NESTED \| FIELD \| CONSTR \| METHOD | DETAIL: FIELD \| CONSTR \| METHOD |  |


---
